# Endoscopic Bariatric Therapies in Inflammatory Bowel Disease: Patient Selection, Nutritional Risk, and Periprocedural Management

**DOI:** 10.3390/metabo16080536

**Published:** 2026-07-29

**Authors:** Paul Grama, Ilie Marius Ciorba, Ștefan Lucian Popa, Abdulrahman Ismaiel, Simona Bataga

**Affiliations:** 1 M3 Department, Discipline of Internal Medicine 1, George Emil Palade University of Medicine, Pharmacy, Science, and Technology of Târgu Mureș, 540139 Târgu Mureș, Romania; paul.grama@umfst.ro (P.G.);; 2 Doctoral School, George Emil Palade University of Medicine, Pharmacy, Science, and Technology of Târgu Mureș, 540139 Târgu Mureș, Romania; 3 2nd Medical Department, “Iuliu Hatieganu” University of Medicine and Pharmacy, 400374 Cluj-Napoca, Romania

**Keywords:** inflammatory bowel disease, obesity, endoscopic bariatric therapy, endoscopic sleeve gastroplasty, intragastric balloon, Crohn’s disease, ulcerative colitis, nutritional risk, sarcopenic obesity, bariatric endoscopy

## Abstract

**Background/Objectives**: Obesity is increasingly recognised as a clinically relevant comorbidity in patients with inflammatory bowel disease (IBD), with potential effects on inflammatory burden, treatment response, surgical risk, nutritional status, and quality of life. Endoscopic bariatric therapies (EBTs), including intragastric balloons and endoscopic sleeve gastroplasty, offer minimally invasive weight-loss options between pharmacotherapy and bariatric surgery. However, their role in IBD remains poorly defined. This narrative review examines patient selection, nutritional risk, periprocedural management, and future research priorities for EBTs in IBD. **Methods**: A narrative review was performed, focusing on obesity in IBD, bariatric endoscopy, bariatric surgery, nutritional deficiencies, sarcopenic obesity, biologic therapy, and periprocedural risk. Evidence from general bariatric endoscopy studies, IBD-specific obesity literature, clinical guidelines, and expert consensus documents was synthesised. **Results**: Direct evidence evaluating EBTs in patients with IBD is very limited. Most practical considerations must therefore be extrapolated from general bariatric endoscopy studies and bariatric surgery data in IBD. Patients with sustained clinical and preferably endoscopic remission, stable nutritional status, and uncomplicated disease phenotypes may represent the most suitable candidates. Active upper gastrointestinal Crohn’s disease, stricturing or penetrating disease, recent surgery, severe malnutrition, sarcopenia, and high-dose corticosteroid exposure should prompt caution or avoidance. Structured follow-up is needed to monitor nutritional deficiencies, body composition, procedural symptoms, and possible IBD activity. **Conclusions**: EBTs may represent a promising option for selected patients with IBD and obesity, but current evidence remains insufficient for firm recommendations. Prospective registries, phenotype-stratified cohort studies, and comparative trials are needed.

## 1. Introduction

### 1.1. Obesity in Inflammatory Bowel Disease

The epidemiological landscape of inflammatory bowel disease (IBD) has undergone a notable transformation over the past two decades, with obesity emerging as an increasingly prevalent comorbidity among patients with both Crohn’s disease (CD) and ulcerative colitis (UC). Historically, IBD, particularly CD, was associated with malnutrition and underweight status. Contemporary data, however, shows that a substantial proportion of patients with IBD now present as overweight or obese, mirroring trends observed in the general population [1].

This paradigm shift has been attributed to multiple converging factors, including the widespread adoption of Western dietary patterns, improved disease control with modern pharmacotherapy, and the rising baseline prevalence of obesity in the general population [2,3]. Gold and Kornbluth have emphasised that obesity is associated with dysbiosis, specifically an increased ratio of Firmicutes to Bacteroidetes, leading to enhanced energy harvest from food and a heightened risk of several gastrointestinal conditions, including IBD [4]. A systematic review and meta-analysis by Camilleri and El-Omar further noted that the direction of the association between obesity and IBD is nuanced and may differ by disease subtype as obesity was associated with a reduced risk of UC but not of CD, while bariatric surgery itself may paradoxically increase the risk of de novo IBD [3]. These complex and sometimes contradictory epidemiological observations underscore the need for a more granular understanding of the obesity-IBD interface.

The recognition that obesity and IBD frequently coexist has prompted calls for integrated management strategies. Ghusn et al. have articulated that obesity represents a state of chronic inflammation that may not only contribute to IBD pathogenesis but also influence disease progression, complications, and response to treatment [2]. The joint European Society for Clinical Nutrition and Metabolism (ESPEN) and United European Gastroenterology (UEG) guideline on obesity care in patients with gastrointestinal and liver diseases has formally acknowledged that patients with IBD should be screened for nutritional status including being overweight and obese at the time of diagnosis and regularly thereafter [5]. This evolving recognition demands that clinicians caring for patients with IBD adopt a proactive stance toward identifying and managing concurrent obesity.

This narrative review was based on literature searches in PubMed, Scopus, and Google Scholar using combinations of the terms inflammatory bowel disease, Crohn’s disease, ulcerative colitis, obesity, endoscopic bariatric therapy, endoscopic sleeve gastroplasty, intragastric balloon, bariatric surgery, nutritional deficiency, sarcopenic obesity, biologic therapy, and periprocedural management. Priority was given to guidelines, consensus statements, systematic reviews, meta-analyses, randomised trials, large cohort studies, and recent narrative reviews. Because direct evidence on EBTs in IBD was scarce, indirect evidence from bariatric endoscopy and bariatric surgery literature was included and clearly identified as extrapolated. A structured narrative approach was deliberately adopted in preference to a systematic review or meta-analysis: because no study has directly evaluated EBTs in a defined IBD population, a quantitative evidence synthesis was not feasible, and a narrative format was better suited to integrating heterogeneous indirect evidence drawn from the bariatric endoscopy, bariatric surgery, and IBD studies. The search covered the period from database inception to April 2026, and was restricted to English-language publications with available full text. Records were screened by title and abstract for relevance to obesity, IBD, and endoscopic or surgical weight-loss interventions; potentially relevant full texts were retrieved, and the reference lists of key articles were hand-searched to identify additional sources. When several sources addressed the same point, the most recent and highest-level evidence was prioritised. Because study selection in a narrative review is inherently susceptible to selection bias, this risk was mitigated by including guidelines and consensus documents alongside primary studies and by explicitly labelling extrapolated evidence throughout the manuscript. Given the small number and observational nature of the IBD-specific sources, a formal PRISMA flow diagram and risk-of-bias appraisal were not applicable to this narrative synthesis.

### 1.2. Why Obesity Matters Clinically in IBD

Obesity in IBD carries clinical significance well beyond its metabolic consequences, with visceral adiposity in particular carrying negative prognostic implications for disease course, response to medical therapy, and surgical risk [2]. Because each of these dimensions is examined in detail in Section 2, they are only outlined here to establish why weight management is a clinically relevant question in this population rather than a purely cosmetic or metabolic one [5].

### 1.3. Current Obesity Treatment Options in IBD

The management of obesity in patients with IBD follows a stepwise, multidisciplinary approach that ascends from lifestyle and dietary modification through anti-obesity pharmacotherapy to bariatric surgery, with endoscopic bariatric therapies (EBTs) occupying an intermediate position between drug therapy and surgery [6]. Each of these tiers, and the IBD-specific considerations that qualify it, is considered in detail in Section 3; the endoscopic options are compared in Table 1.

### 1.4. Why Endoscopic Bariatric Therapies Deserve a Dedicated Discussion in IBD

Despite the growing recognition that EBTs occupy a valuable niche in the obesity treatment armamentarium, their application in patients with IBD has received remarkably little dedicated attention. Ghusn et al. acknowledged that endoscopic bariatric therapies and even bariatric surgery may be effective and well tolerated in selected patients with IBD, yet the evidence base remains sparse and largely extrapolated from general population studies [2]. Storm et al., in their foundational primer on endobariatrics, listed upper gastrointestinal IBD as a relative contraindication to IGB therapy, but provided no further guidance on how to evaluate, prepare, or monitor patients with IBD who might otherwise be candidates for endoscopic procedures [7]. The ESPEN/UEG guideline addressed obesity management in IBD comprehensively with respect to nutritional screening, bariatric surgery, and long-term follow-up, yet offered no specific recommendations regarding the use of EBTs in this population [5].

Several features of IBD render the application of EBTs uniquely challenging: active mucosal inflammation, strictures, fistulae, or prior intestinal resections may alter the safety profile of intragastric devices and endoscopic suturing [7]; a heightened baseline risk of nutritional deficiency may be exacerbated by the restrictive or malabsorptive mechanisms of EBTs [5], a concern compounded by how little the nutritional consequences of non-surgical endoscopic procedures have been studied [8]; immunosuppressive and biologic therapies may influence wound healing and infection risk; and the need for rigorous evaluation, multidisciplinary care, and guaranteed nutritional support is amplified where concurrent disease monitoring is required [9]. Each of these is examined in detail in Section 5.

The European Society of Gastrointestinal Endoscopy (ESGE) Position Statement on bariatric endoscopy training has outlined general contraindications and patient selection criteria for EBTs, including the need for multidisciplinary team evaluation, but does not specifically address the IBD population [10]. Similarly, comprehensive reviews of EBTs have catalogued the available devices, their mechanisms, efficacy, and adverse event profiles in the general population [11,12,13,14]. However, no dedicated review has provided a practical synthesis focused on patient selection, nutritional risk, and periprocedural management in patients with IBD. This review therefore summarises the current evidence, identifies clinically relevant gaps, and proposes practical considerations for the use of EBTs in carefully selected patients with IBD and obesity.

## 2. Obesity as a Disease Modifier in Inflammatory Bowel Disease

### 2.1. Effects of Obesity on Inflammatory Burden and Disease Phenotype

The biological interplay between obesity and IBD involves mechanistic pathways centred on visceral adipose tissue, mesenteric fat, and adipokine signalling. Visceral adipose tissue is a metabolically and immunologically active organ secreting pro-inflammatory cytokines, including TNF-α, IL-6, and IL-1β, alongside adipokines such as leptin, resistin, and visfatin, all implicated in amplifying intestinal inflammation. In CD, “creeping fat”, the hypertrophic expansion of mesenteric adipose tissue encasing inflamed intestinal segments, has been a recognised hallmark since the original description of Crohn’s [1,15,16,17,18]. It is characterised by adipocyte hyperplasia rather than hypertrophy, with a roughly four-fold increase in mesenteric adipocyte number compared with healthy controls, and correlates positively with transmural inflammation, fibrosis, stricture formation, and perivascular macrophage and lymphocyte infiltration. The interaction between an inflamed gut wall and visceral fat may not only sustain but amplify inflammation in a pathogenic feedback loop, though whether adipose inflammation drives intestinal disease or results from it remains unclear [1]. Bacterial translocation into mesenteric adipose tissue has been demonstrated, with Proteobacteria the most abundant phylum in creeping fat and their abundance correlating with faecal calprotectin and C-reactive protein, while leptin, overexpressed in mesenteric fat, promotes macrophage activation, Th1 and Th17 responses, and NF-κB activation in intestinal epithelial cells [1,15,18,19,20]. Altered adipose biology has also been reported in UC, including oedematous adipose tissue and enlarged mesenteric lymph nodes, albeit less consistently [16,21]. Visceral and mesenteric fat are therefore active participants in the inflammatory milieu of IBD, with the potential to modify disease phenotype and behaviour.

From a metabolic standpoint, these adipose-derived mediators position obesity as an active metabolic disease modifier in IBD rather than a passive comorbidity. The adipokines secreted by visceral and mesenteric fat—particularly leptin, resistin, and visfatin—act not only as pro-inflammatory signals but also as metabolic regulators linking energy balance, insulin sensitivity, and innate immune activation, while the relative deficiency of anti-inflammatory adiponectin that characterises visceral obesity may further tilt this balance toward a pro-inflammatory, insulin-resistant phenotype. In parallel, obesity-associated dysbiosis, notably the increased Firmicutes-to-Bacteroidetes ratio and the enrichment of Proteobacteria within creeping fat-alters the output of microbiota-derived metabolites and enhances energy harvest, providing a mechanistic bridge between the gut microbiome, mesenteric adiposity, and intestinal inflammation. These observations suggest that weight-loss interventions, including EBTs, may influence IBD through effects that extend beyond a reduction in body weight to encompass shifts in adipokine signalling, the microbiota–metabolite axis, and systemic metabolic inflammation—a hypothesis that remains untested in IBD-specific cohorts [22,23,24].

### 2.2. Obesity and Response to Medical Therapy

A growing body of evidence suggests that obesity adversely influences the efficacy of medical therapies in IBD, particularly biologic agents. A study of 160 patients with UC treated with biologics demonstrated that each 1 kg/m^2^ increase in BMI was associated with an increased risk of treatment failure and surgery or hospitalisation of up to 4% and 8%, respectively. However, the evidence is not uniformly consistent: an analysis of 1206 subjects with IBD (both CD and UC) did not demonstrate significant differences in response to infliximab between obese and non-obese patients [25]. Sun et al. have similarly emphasised that obesity has been demonstrated to be associated with an attenuated response to immunomodulators and biological agents, as well as higher rates of perioperative surgical complications [22]. These discrepancies may reflect the limitations of BMI as a measure of adiposity, differences in study populations, or the heterogeneity of IBD phenotypes. Nonetheless, the preponderance of evidence supports the conclusion that excess adiposity—particularly visceral adiposity—is a clinically relevant modifier of treatment response in IBD.

### 2.3. Obesity and Risk of Hospitalisation, Surgery, and Postoperative Complications

Beyond its effects on medical therapy, obesity has been associated with increased healthcare utilisation and surgical risk in IBD. Readmissions can appear in IBD depending on multiple risk factors [26]. The ESPEN/UEG guideline recommends that screening for nutritional status and, if indicated, comprehensive nutritional assessment be performed in patients with IBD and obesity before intestinal surgery to identify the need for perioperative nutritional therapy [5]. Obesity compounds perioperative risk through multiple mechanisms, including increased technical difficulty of surgery, impaired wound healing, and a heightened risk of infectious complications. Sun et al. noted that obesity is associated with higher rates of perioperative surgical complications in IBD [22]. Kaazan et al. further observed that the association of creeping fat with expanding mesenteric adipose tissue, transmural inflammation, and a thickened intestinal wall may correlate with a more complex disease process and increased surgical morbidity in CD [21]. These findings collectively establish that obesity is a significant modifier of surgical risk and postoperative outcomes in IBD, with implications for both preoperative optimisation and the selection of weight management strategies.

### 2.4. Sarcopenic Obesity and the Limitations of BMI

A critical limitation of much of the existing literature is reliance on body mass index (BMI) as the primary measure of adiposity. BMI distinguishes neither subcutaneous from visceral fat nor lean body mass, and a formally adequate BMI may mask increased body fat with low muscle mass, a condition termed sarcopenic obesity that correlates with higher rates of rehospitalisation during flares and poorer clinical outcomes [25]. Sarcopenia, the pathological loss of skeletal muscle mass and function, is highly prevalent in IBD and may develop through malnutrition, chronic inflammation, vitamin deficiency, and imbalance of the muscle–gut axis; its coexistence with excess adiposity is particularly insidious because a normal or elevated BMI leads to under-recognition of nutritional risk. The ESPEN/UEG guideline accordingly recommends that weight-loss therapy in patients with IBD and obesity consist of fat reduction without muscle loss [5]. Visceral adiposity assessed by cross-sectional imaging is a more reliable marker of obesity-related complications in CD than BMI alone, and the mesenteric fat index has been correlated with postoperative recurrence and disease activity [27]. Body-composition assessment beyond BMI is therefore needed when evaluating IBD patients with obesity, particularly before interventions that may differentially affect fat and lean mass [28].

## 3. Current Weight-Management Strategies in Inflammatory Bowel Disease

### 3.1. Lifestyle and Dietary Approaches

Lifestyle intervention (diet, physical activity, and behavioural counselling) is the foundational tier of obesity management, typically via a 500–700 kcal/day deficit [6]. Structured lifestyle programmes typically yield a modest 3–5% TBWL, with frequent regain [29]. In patients with IBD, however, the implementation of lifestyle interventions is complicated by several disease-specific factors. Fatigue, abdominal pain, diarrhoea, and the psychological burden of chronic illness may limit adherence to exercise regimens and dietary plans.

### 3.2. Pharmacologic Anti-Obesity Therapies

Incretin-based therapies, particularly GLP-1 receptor agonists (GLP-1 RAs) and dual GLP-1/GIP agonists, have transformed obesity pharmacotherapy [1], with semaglutide achieving roughly 15–17% TBWL and tirzepatide more [3]. Beyond their metabolic effects, GLP-1 RAs have demonstrated anti-inflammatory properties, including modulation of nuclear factor kappa-light-chain-enhancer of activated B cells (NF-κB) signalling, reduction in pro-inflammatory cytokines, and potential improvement of intestinal barrier integrity, raising the possibility that these agents may confer disease-specific benefits in IBD [1]. Ghusn et al. noted that anti-obesity medications may be effective and well tolerated in selected patients with IBD, although data specific to this population remain limited and largely derived from case series or retrospective analyses [2]. Deza et al. similarly highlighted the potential utility of metabolic drugs such as GLP-1 RAs in IBD management, while cautioning that prospective studies are warranted to elucidate their clinical significance in this context [1].

Despite these promising signals, several concerns temper enthusiasm for pharmacotherapy in IBD. GLP-1 RAs are associated with gastrointestinal side effects-including nausea, vomiting, diarrhoea, and delayed gastric emptying-that may overlap with or exacerbate IBD symptoms, complicating clinical assessment. Weight regain after drug withdrawal is well documented, necessitating long-term or indefinite treatment. Additionally, the interaction between anti-obesity pharmacotherapy and the immunosuppressive or biologic agents commonly used in IBD has not been systematically studied, leaving important safety questions unanswered.

### 3.3. Bariatric Surgery

Bariatric surgery (sleeve gastrectomy and Roux-en-Y gastric bypass, RYGB) remains the most effective intervention for severe obesity [6]. In patients with IBD, a growing body of evidence suggests that bariatric surgery is overall safe and effective. Ghusn et al. reported that a case–control study found patients with IBD who underwent bariatric surgery not only achieved significant BMI reduction but also experienced less frequent IBD-related surgeries and corticosteroid use compared to nonsurgical counterparts. However, important nuances exist: patients with CD have been reported to have higher readmission rates post-surgery, and patients with UC may face more serious complications after RYGB but not after SG. Camilleri and El-Omar further noted that bariatric surgery itself may paradoxically increase the risk of de novo IBD, an observation that warrants further investigation [3].

Bariatric surgery, particularly malabsorptive RYGB, carries a well-established risk of micronutrient deficiencies (iron, vitamin B12, vitamin D, calcium, protein), which are compounded in IBD, where malabsorption and nutritional deficiency are already prevalent. The ESPEN/UEG guideline recommends that all patients undergoing bariatric surgery, including those with chronic gastrointestinal diseases, should be evaluated for nutritional deficiencies and sarcopenia before intervention [5,8]. The Polish Expert Consensus on Metabolic and Bariatric Surgery has further emphasised that regular bariatric and gastroenterological follow-up is crucial in managing additional problems arising from IBD and should be included in the postoperative care plan [30]. Bariatric surgery is irreversible and carries perioperative risks that may be amplified in IBD.

### 3.4. Where Endoscopic Bariatric Therapies Fit in the Treatment Pathway

Endoscopic bariatric therapies (EBTs) occupy an intermediate position between pharmacotherapy and surgery, offering greater efficacy than medical therapy while remaining less invasive, potentially reversible, and repeatable relative to surgery [7,11,31,32]. They may therefore serve either as a bridge to surgery in severe obesity or as a primary intervention in class I–II obesity that does not meet surgical eligibility criteria [12,31]. The individual modalities, their mechanisms, and the strength of their evidence base are described in Section 4 and summarised in Table 1.

### 3.5. Why a Tailored Approach Is Needed in IBD

The selection of an appropriate weight management strategy in patients with IBD cannot be guided solely by BMI thresholds or general obesity treatment algorithms. Several IBD-related factors must be integrated into clinical decision-making. First, disease activity-including the presence of active mucosal inflammation, strictures, fistulae, or prior intestinal resections-may alter the safety profile and feasibility of both surgical and endoscopic interventions [2,21]. Second, nutritional status must be carefully assessed, as patients with IBD are at heightened baseline risk for protein-calorie malnutrition, micronutrient deficiencies, and sarcopenia, all of which may be exacerbated by restrictive or malabsorptive bariatric procedures [25]. Third, the immunosuppressive and biologic therapies commonly used in IBD may influence wound healing, infection risk, and procedural outcomes in ways that have not been systematically studied in the context of bariatric interventions. Fourth, the psychological burden of living with a chronic gastrointestinal disease may affect adherence to postprocedural dietary regimens and follow-up protocols.

## 4. Overview of Endoscopic Bariatric Therapies

### 4.1. Definition and Classification of Endoscopic Bariatric Therapies

Endoscopic bariatric therapies (EBTs) constitute a heterogeneous group of minimally invasive endoluminal procedures designed to induce weight loss and improve obesity-related metabolic comorbidities through gastric volume reduction, space occupation, delayed gastric emptying, altered nutrient absorption, and hormonal modulation [8,11,33]. The American Society of Gastrointestinal Endoscopy (ASGE) has recommended that EBTs achieve a mean threshold of greater than 5% total body weight loss (TBWL), greater than 25% excess weight loss (EWL), and a risk of serious adverse events below 5% [13,14]. They may be broadly divided into gastric procedures—space-occupying devices (intragastric balloons), aspiration therapy, and gastric remodelling techniques (endoscopic sleeve gastroplasty, POSE)—and small-bowel interventions such as the duodenal–jejunal bypass liner and duodenal mucosal resurfacing [8,34,35]. The evidence base is strongest for intragastric balloons and endoscopic sleeve gastroplasty, which have been evaluated in randomised controlled trials and large observational cohorts; whereas, data for the other modalities remain more limited.

### 4.2. Intragastric Balloons

Intragastric balloons (IGBs) are the longest-established EBTs; these space-occupying devices reduce functional gastric volume, promote early satiety, and delay gastric emptying [8,11,31,36]. Several IGB systems have regulatory approval, differing in fill material, volume, and approved placement duration (typically 6–12 months) [36,37]. A newer swallowable, self-deflating balloon does not require endoscopy for placement or removal [12,38].

IGBs typically achieve about 10–15% TBWL at 6 months, with efficacy figures summarised in Table 1 [13,14,39]. Weight regain after removal is common, and early nausea, vomiting, and abdominal pain occur frequently [12,40]. Serious complications (ulceration, migration, obstruction, perforation) are rare, and IGBs retain a role for class I–II obesity and as a bridge to surgery [41]. For IBD, the reversible nature of IGBs is attractive, but the high incidence of gastrointestinal symptoms and the requirement for intact gastric mucosa are clinically relevant considerations discussed in later sections.

### 4.3. Endoscopic Sleeve Gastroplasty

Endoscopic sleeve gastroplasty (ESG) is the most widely adopted gastric-remodelling EBT and has the strongest evidence base among endoscopic tissue apposition procedures [14,33,39,42]. It uses endoscopic full-thickness suturing along the greater curvature to reduce gastric volume by approximately 70%, mimicking laparoscopic sleeve gastrectomy (LSG) without incisions [34,43,44].

In the randomised MERIT trial, ESG achieved a mean EWL of 49.2% versus 3.2% for lifestyle control at 52 weeks [45]. Meta-analyses report TBWL of 15–20% at 12 months, sustained to 24 months (Table 1) [14,44]. ESG yields greater, more durable weight loss than IGBs though less than LSG, with a favourable safety profile (serious adverse events ~2–4%) [46,47,48].

### 4.4. Primary Obesity Surgery—Endoluminal and Related Plication Techniques

The Primary Obesity Surgery Endoluminal (POSE) procedure is an alternative gastric-plication technique that reduces gastric volume and promotes early satiety; the more recent POSE 2.0 targets the gastric body and antrum [49]. Pooled TBWL is approximately 12.7–13.5%, with efficacy summarised in Table 1 [2,46,49,50].

The evidence base for POSE is considerably smaller than for ESG, which achieved superior EWL in a comparative meta-analysis [35]. POSE is not FDA-approved for bariatric use and is less widely adopted; its more limited evidence base is relevant when considering complex populations such as those with IBD [46,49,51].

The principal endoscopic bariatric therapies, their mechanisms, reported efficacy in the general obesity population, evidence base, and the considerations that are specific to patients with IBD are summarised in Table 1.

## 5. Why Inflammatory Bowel Disease Changes the Discussion Around Endoscopic Bariatric Therapies

### 5.1. Chronic Intestinal Inflammation and Systemic Consequences

The fundamental distinction between patients with IBD and the general obesity population lies in the presence of chronic, relapsing intestinal inflammation with far-reaching systemic consequences. IBD is characterised by dysregulated immune responses producing cycles of mucosal injury and repair, driven by pro-inflammatory cytokines including TNF-α, IL-6, and IL-1β secreted not only by immune cells within the gut wall but also by metabolically active visceral and mesenteric adipose tissue [21,22]; the Western lifestyle and diet further fuel metabolic inflammation in and beyond the gut, and metabolic disorders increasingly affect patients with IBD with an expected negative impact on both disease entities [52]. Active inflammation elevates C-reactive protein, faecal calprotectin, and circulating cytokines, any of which may impair mucosal healing after endoscopic procedures and increase the risk of bleeding, perforation, or delayed tissue repair, and the systemic inflammatory state may additionally alter the pharmacokinetics of sedative and analgesic agents, though this has not been formally studied. Safety and efficacy data generated in metabolically healthy obese populations therefore cannot be directly extrapolated to patients with active or recently active IBD, and disease-activity assessment must precede any endoscopic bariatric intervention.

### 5.2. Prior Bowel Surgery and Altered Anatomy

A substantial proportion of patients with IBD, particularly those with CD, will require intestinal surgery during their disease course; stricturing (B2) or penetrating (B3) behaviour under the Montreal classification is associated with disease severity and the need for surgery [53]. Prior ileocecal or segmental small-bowel resection, colectomy, or ileal pouch-anal anastomosis fundamentally alters gastrointestinal anatomy and physiology in ways directly relevant to EBT selection and safety. Patients with prior extensive small-bowel resection may already have a shortened absorptive surface, rendering any additional malabsorptive intervention potentially hazardous [2]. This applies with particular force to the small-bowel-targeted devices: the duodenal–jejunal bypass liner and duodenal mucosal resurfacing are deployed in the proximal small bowel and are especially problematic in Crohn’s disease involving the upper gastrointestinal tract or proximal small bowel, where active or stricturing duodenal or jejunal disease may preclude safe deployment, increase the risk of device impaction or perforation, and, for the bypass liner, compound an already malabsorptive state. Small-bowel EBTs are therefore best avoided in patients with known upper-gastrointestinal or proximal small-bowel Crohn’s disease. Altered anatomy after prior surgery may likewise affect the technical feasibility of IGB placement or ESG, as adhesions, anastomotic sites, or diverted segments may preclude safe deployment or suture placement. Bariatric surgery in established IBD is technically feasible but requires careful selection, and the resulting anatomic changes may themselves promote dysbiosis and, in genetically predisposed patients, exaggerated intestinal inflammation [54]. These considerations mandate a thorough review of surgical history and, where indicated, cross-sectional imaging before any endoscopic bariatric procedure is offered.

### 5.3. Strictures, Fistulas, Adhesions, and Obstruction Risk

Phenotype-specific concerns are particularly salient in CD, where stricturing (B2) and penetrating (B3) behaviour directly affect the safety of endoluminal devices. Perianal disease and refractoriness to biologics have been reported more frequently in overweight and obese CD patients, suggesting that the very patients most likely to be considered for bariatric intervention may harbour the most complex phenotypes [55]. Strictures, whether inflammatory, fibrotic, or mixed, pose a risk of device impaction, balloon migration, or obstruction should an intragastric balloon deflate and pass distally. Fistulising disease introduces the hazard of creating or worsening abnormal communications between bowel segments, the peritoneal cavity, or adjacent organs during endoscopic manipulation, while adhesions from prior surgery or transmural inflammation may tether bowel loops and increase the risk of perforation during full-thickness suturing such as ESG. The association of creeping fat with expanding mesenteric adipose tissue, transmural inflammation, and a thickened intestinal wall may correlate with a more complex disease process and increased procedural morbidity [21]. These risks underscore the necessity of comprehensive disease staging, including cross-sectional imaging and, where appropriate, small-bowel evaluation, before EBT is considered in any patient with CD.

### 5.4. Malabsorption and Micronutrient Vulnerability

Patients with IBD are at heightened baseline risk for malabsorption and micronutrient deficiency, a vulnerability compounded by the restrictive or malabsorptive mechanisms of many EBTs. Deficiencies in iron, vitamin B12, folate, vitamin D, calcium, zinc, and protein are common, with pattern and severity varying by disease location, extent, and prior surgical history, and arise through decreased intake, chronic inflammation, malabsorption, and the effects of pharmacological treatment [22]; hypermetabolism and long-term parenteral nutrition may compound this further [2]. Introducing an endoscopic bariatric device may precipitate or worsen clinically significant deficiency, with consequences ranging from anaemia and osteoporosis to neurological impairment. The risk of sarcopenic obesity is particularly relevant, as weight loss that does not preserve muscle mass may paradoxically worsen functional status and disease outcomes. These vulnerabilities demand rigorous pre-procedural nutritional assessment, individualised supplementation, and structured post-procedural monitoring, as detailed in Section 7 and Section 8.

### 5.5. Corticosteroids, Biologics, and Immunomodulators

The pharmacological landscape of IBD introduces further complexity. Corticosteroids, still widely used for induction of remission, impair wound healing, increase infection risk, and cause adrenal suppression and metabolic derangement; chronic exposure may compromise gastric wall integrity, increasing the risk of perforation or suture dehiscence during procedures such as ESG. Steroid treatment, reduced physical activity from fatigue, and poor general condition may together alter body composition [55]. Biologics, including anti-TNF agents, vedolizumab, and ustekinumab, modulate immune function in ways that may influence procedural healing and infection risk, though these interactions remain unstudied in the EBT setting; notably, patients on anti-TNF combination therapy or vedolizumab monotherapy are more likely to be overweight or obese, so the very medications used to control IBD may enlarge the pool of candidates for bariatric intervention [56]. Thiopurines and methotrexate compound immunosuppression and may affect hepatic metabolism and haematologic parameters relevant to procedural safety. The timing of EBTs relative to immunosuppressive therapy is an important but currently unaddressed aspect of peri-procedural planning, considered further in Section 7.5.

### 5.6. Symptom Overlap Between Procedural Effects and IBD Activity

The expected side effects of EBTs overlap substantially with the symptoms of active IBD. Nausea, vomiting, abdominal pain, bloating, early satiety, and altered bowel habits are among the most common adverse effects after IGB placement and ESG, and are equally cardinal features of an IBD flare, making a benign procedural effect difficult to distinguish from a clinically significant exacerbation [22]. The consequences of that ambiguity, and the monitoring protocols required to resolve it, are addressed in Section 7.8 [52].

## 6. Patient Selection for Endoscopic Bariatric Therapy in Inflammatory Bowel Disease

### 6.1. Which Patients May Benefit Most

**Figure 1:**
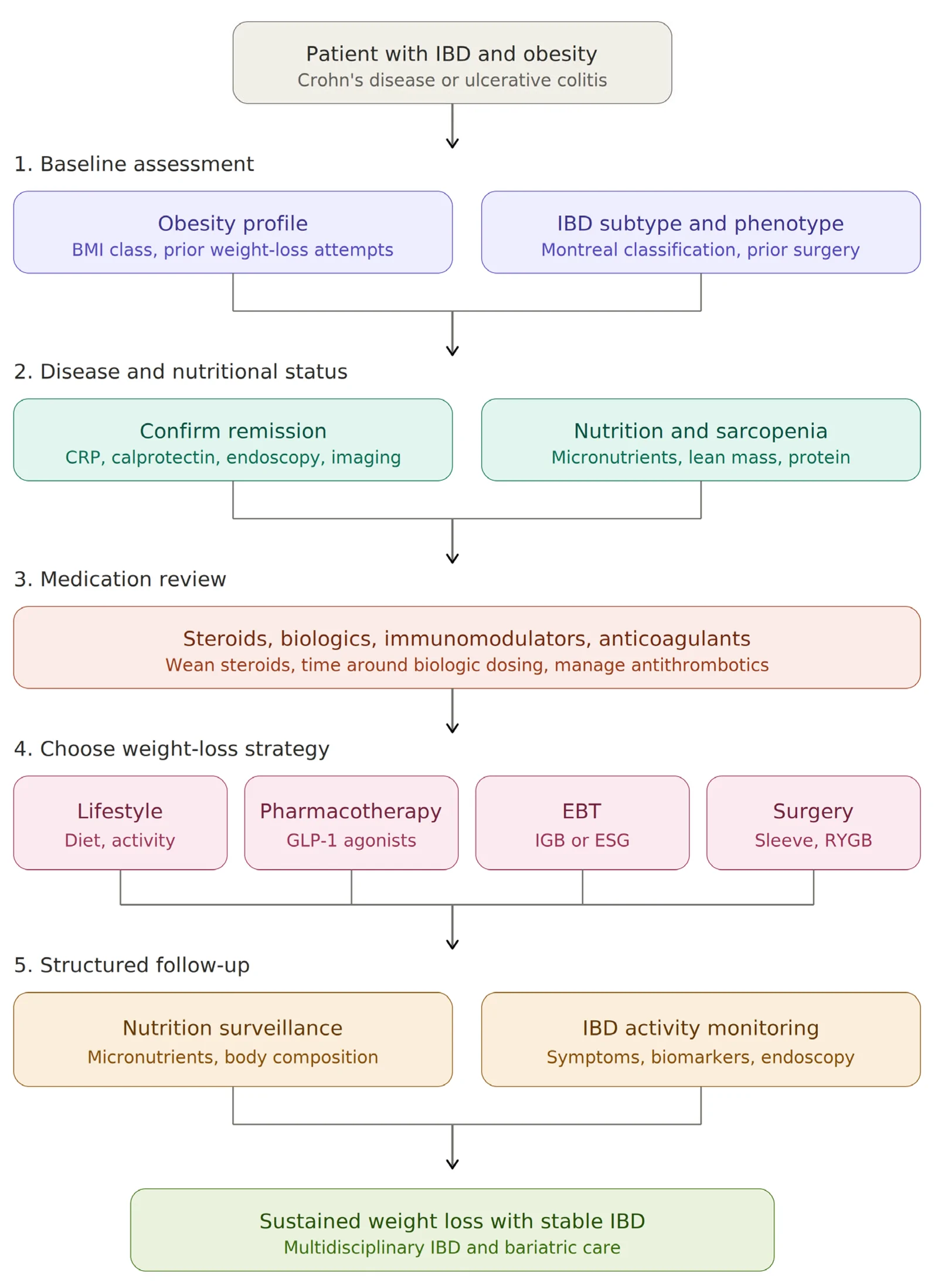
Proposed multidisciplinary pathway for the assessment and selection of weight-loss strategies in patients with inflammatory bowel disease (IBD) and obesity. The pathway integrates obesity profile, IBD subtype and phenotype, disease activity, nutritional status, medication review, treatment selection, and structured follow-up. Endoscopic bariatric therapies (EBTs), including intragastric balloon (IGB) placement and endoscopic sleeve gastroplasty (ESG), should be considered only after individualised assessment of disease remission, nutritional risk, prior surgery, medication exposure, and follow-up capacity. This algorithm is proposed as a practical clinical framework based on indirect evidence and expert reasoning, and requires prospective validation in IBD-specific cohorts.

A proposed multidisciplinary pathway for weight-loss strategy selection in patients with IBD and obesity is presented in Figure 1. None of the branch points in this pathway is supported by direct, IBD-specific evidence for EBTs; each reflects either extrapolation from bariatric surgery and general endoscopic practice or expert consensus, as detailed and labelled in Table 2. The algorithm is therefore a structured framework for organising multidisciplinary decision-making rather than a validated decision rule, and requires prospective validation in IBD-specific cohorts before it can be regarded as evidence-based. In the absence of prospective trials, patient selection must synthesise general bariatric endoscopy eligibility criteria, IBD-specific considerations, and expert reasoning. The patients most likely to benefit are those with well-controlled IBD and class I or II obesity (BMI 30–40 kg/m^2^) who have failed or cannot sustain adequate weight loss with lifestyle measures and pharmacotherapy, and who either do not meet criteria for or decline bariatric surgery [2,22]. A large propensity score–matched cohort found bariatric surgery to be associated with improved IBD-related outcomes among patients with concomitant obesity and IBD, suggesting that weight reduction itself may confer disease-specific benefit and supporting the rationale for less invasive weight-loss interventions in appropriately selected patients [57]. Patients in whom obesity is clearly contributing to impaired treatment response, increased surgical risk, or diminished quality of life represent a particularly compelling subgroup.

### 6.2. Disease Remission Versus Active Disease

The distinction between quiescent and active IBD is arguably the single most important determinant of candidacy. Active mucosal inflammation compromises tissue integrity, impairs wound healing, and increases the risk of bleeding, perforation, or device-related mucosal injury, while the systemic inflammatory state may alter sedative pharmacokinetics and increase post-procedural infectious risk. Extrapolating from the bariatric surgery literature and general endoscopic principles, EBTs should therefore be offered only to patients in sustained clinical and, ideally, endoscopic remission; disease stability at the time of intervention was likewise central to the matching strategy of the largest available cohort [57]. The STRIDE-II criteria, defining clinical remission, biomarker normalisation, and endoscopic healing, provide a useful framework for determining procedural readiness [58,59]. Patients with recent flares, elevated faecal calprotectin or C-reactive protein, or endoscopic evidence of active ulceration should be considered ineligible until disease control has been re-established and maintained—a threshold not formally defined for EBTs, but reasonably at least three to six months of documented remission.

### 6.3. Crohn’s Disease Versus Ulcerative Colitis

The two major subtypes present distinct considerations. Ulcerative colitis, confined to the colonic mucosa, does not directly involve the upper gastrointestinal tract or small bowel, the primary anatomical targets of most EBTs; patients with UC in remission may therefore be more straightforward candidates for gastric-based procedures, provided no concurrent upper gastrointestinal pathology is present. Patients with UC have been found to experience a lower risk of IBD-related hospitalisation and reduced systemic corticosteroid use following bariatric surgery, suggesting weight-loss interventions may be particularly beneficial in this subgroup [57], although the anatomic changes secondary to bariatric procedures may themselves promote dysbiosis and, in genetically predisposed patients, exaggerated intestinal inflammation [54].

Crohn’s disease, by contrast, introduces substantially greater complexity. CD can involve any segment of the gastrointestinal tract from the mouth to the anus, and upper gastrointestinal involvement (including gastric and duodenal disease) has been reported in a significant minority of patients [60]. The presence of gastric or duodenal Crohn’s disease would represent a relative or absolute contraindication to most gastric-based EBTs, as device placement or suturing in inflamed tissue carries potential increased risk. Furthermore, the transmural nature of CD inflammation, the propensity for stricture and fistula formation, and the frequent need for intestinal resection all introduce additional hazards that are largely absent in UC [54].

### 6.4. Impact of Disease Phenotype, Including Stricturing, Penetrating, Perianal, and Postoperative Disease

Disease phenotype under the Montreal classification is a critical determinant of candidacy in CD, since stricturing (B2) or penetrating (B3) behaviour is associated with disease severity and the need for surgery [53], and perianal disease and biologic refractoriness are more frequent in overweight and obese CD patients [55]. The device-related hazards these phenotypes create—impaction or obstruction at a strictured segment, particularly where a deflated intragastric balloon migrates distally, and the worsening of fistulous communications during endoscopic manipulation—are set out in Section 5.3 and summarised in Table 2 [2,54]. Perianal disease, while not a direct contraindication to gastric-based EBTs, may signal a more aggressive phenotype, and postoperative recurrence at anastomotic sites warrants particular caution given subclinical inflammation at surgical junctions. Patients with inflammatory (B1) behaviour, limited disease extent, and no history of stricturing or penetrating complications represent the most favourable phenotype for EBT consideration.

### 6.5. Contraindications and Red Flags

No formal contraindication list for EBTs in IBD has been published; the scenarios that should prompt caution or avoidance are set out with their evidentiary basis in Table 2. In brief, active upper gastrointestinal inflammation, including gastric or duodenal Crohn’s disease, is a clear contraindication to gastric-based EBTs, and known strictures distal to the device increase the risk of obstruction [54]. Active fistulising or penetrating disease, uncontrolled perianal disease, and recent intestinal surgery (generally within three to six months) are red flags, while severe malnutrition, sarcopenia, or uncorrected micronutrient deficiency should be addressed before intervention [61]. Current or recent high-dose corticosteroid therapy impairs healing and raises infection risk, and multiple prior abdominal surgeries with extensive adhesive disease increase procedural risk, particularly with full-thickness suturing. Finally, the inability to ensure adequate post-procedural follow-up, including IBD monitoring, nutritional surveillance, and multidisciplinary access, should be regarded as a relative contraindication.

Proposed candidacy criteria, cautions, and red flags for EBTs according to IBD features, together with the level of supporting evidence, are summarised in Table 2. This table makes explicit which considerations rest on indirect or extrapolated evidence and which reflect expert consensus.

## 7. Pre-Procedural Assessment and Peri-Procedural Management of Endoscopic Bariatric Therapies in Patients with Inflammatory Bowel Disease

### 7.1. Clinical History and Phenotype Review

Safe pre-procedural planning for an EBT in IBD rests on a meticulous clinical history and disease phenotype review that goes well beyond the standard bariatric endoscopy work-up. The clinician should document IBD subtype (CD, UC, or IBD-unclassified), disease duration, Montreal classification, history and frequency of flares, prior hospitalisations, and all previous IBD-related surgery [53]. Stricturing (B2) or penetrating (B3) behaviour in CD warrants particular attention, as these phenotypes carry specific risks of obstruction, perforation, and fistula formation. Perianal disease and refractoriness to biologics have been reported more frequently in overweight and obese CD patients, underscoring the value of identifying these features at the initial evaluation [55]. Extraintestinal manifestations, comorbidities (metabolic syndrome, cardiovascular disease, venous thromboembolism risk), and psychosocial factors should also be recorded, as each may influence procedural risk and post-procedural management [10].

### 7.2. Endoscopic and Imaging Assessment When Indicated

Before any gastric-based EBT, upper gastrointestinal endoscopy should evaluate the gastric and duodenal mucosa for active inflammation, ulceration, erosions, or other pathology that would preclude safe device placement or suturing. Upper gastrointestinal IBD has been listed as a relative contraindication to intragastric balloon (IGB) therapy [7], a principle that extends logically to endoscopic sleeve gastroplasty (ESG) and other gastric remodelling procedures, and gastric involvement has separately been treated as a contraindication to IGB placement [62]. In CD, cross-sectional imaging, preferably magnetic resonance or computed tomography enterography, should evaluate small-bowel strictures, fistulae, abscesses, or mesenteric inflammatory change, particularly where a small-bowel-targeted device is contemplated. Large hiatal hernia, portal hypertensive gastropathy, and gastric varices should be excluded as established contraindications to most gastric EBTs [7,13]. No formal evidence-based protocol for pre-procedural imaging in IBD patients undergoing EBTs has been published; the recommendations outlined here are extrapolated from bariatric surgery guidelines and general endoscopic practice and should be interpreted accordingly.

### 7.3. Laboratory Work-Up

A comprehensive laboratory work-up should precede EBT in all patients with IBD: complete blood count, comprehensive metabolic panel including hepatic and renal function, C-reactive protein, erythrocyte sedimentation rate, and faecal calprotectin as objective markers of disease activity [61]. Coagulation studies are warranted in patients on anticoagulant or antiplatelet therapy or with hepatic dysfunction, and glycated haemoglobin (HbA1c) should be measured where metabolic syndrome or diabetes is known or suspected [8,30]. Thyroid function tests may be considered to exclude secondary causes of obesity. The laboratory assessment serves a dual purpose: confirming disease quiescence and establishing a baseline against which post-procedural changes can be compared.

### 7.4. Nutritional Screening and Micronutrient Testing

Nutritional screening is a critical and non-negotiable component of the pre-procedural evaluation. The ESPEN guideline on clinical nutrition in IBD recommends screening and, where indicated, comprehensive nutritional assessment at diagnosis and regularly thereafter, with particular attention to patients undergoing bariatric interventions [61]. Patients with IBD are at heightened baseline risk of deficiency in iron, vitamin B12, folate, vitamin D, calcium, zinc, magnesium, and protein, with pattern and severity varying by disease location, extent, and prior surgical history. The AGA Technical Review on intragastric balloons highlighted thiamine, folate, magnesium, and potassium as essential to evaluate before IGB placement, as these are affected rapidly during the indwelling period [63]; few studies have assessed nutritional deficiencies after non-surgical endoscopic bariatric procedures at all, a gap of particular concern in a population already predisposed to malnutrition [8]. The recommended baseline panel is set out in Table 3, and any identified deficiency should be corrected before the procedure is undertaken. Body-composition assessment should be considered to identify sarcopenic obesity, since weight loss that does not preserve lean mass may paradoxically worsen functional status.

### 7.5. Medication Review, Including Corticosteroids, Biologics, Immunomodulators, and Antithrombotic Therapy

A thorough medication review is essential before proceeding with any EBT in a patient with IBD. Biologic agents, including anti-TNF therapies, anti-integrins, and anti-IL-12/23 agents, modulate immune function in ways that may influence procedural healing and infection risk, although these interactions have not been systematically studied in the context of EBTs [2,56]. With regard to whether biologic therapy should be continued or temporarily held, no IBD-specific evidence exists for the EBT setting. By analogy with elective abdominal surgery, where current IBD guidance generally favours continuing biologic therapy rather than routinely interrupting it, a reasonable default is to maintain established treatment and to schedule the procedure toward the end of the dosing interval, reserving any modification for individualised multidisciplinary discussion. Corticosteroids warrant particular caution, as they are the immunosuppressant most consistently linked to post-procedural infection and impaired healing; where feasible, patients should be weaned to the lowest effective dose or transitioned to steroid-sparing therapy before a full-thickness procedure such as ESG, and those on chronic corticosteroids may require perioperative stress-dose coverage to mitigate the risk of adrenal insufficiency. Immunomodulators such as thiopurines and methotrexate should be reviewed for their effects on haematologic parameters and hepatic function [55]. Antithrombotic therapy, including anticoagulants and antiplatelet agents, must be managed according to established peri-procedural guidelines for therapeutic endoscopy, balancing the risk of bleeding against that of thromboembolic events, which is particularly relevant for ESG given its full-thickness suturing [10,64]. Nonsteroidal anti-inflammatory drugs should be discontinued prior to IGB placement, as their use has been associated with gastric ulcer formation in the presence of intragastric devices [34,45].

### 7.6. Procedural Planning and Anaesthesia Considerations

Procedural planning should involve close collaboration between the bariatric endoscopist, the IBD specialist, the anaesthesiologist, and the nutritional support team. ESG is typically performed under general anaesthesia, while IGB placement and removal may be performed under monitored anaesthesia care, although retained gastric contents may necessitate endotracheal intubation for airway protection during removal. Obesity alters airway management, positioning, and the pharmacokinetics of anaesthetic agents, and each EBT carries specific nuances with important implications for the anaesthesiologist [65]. In IBD, additional considerations include potential adrenal insufficiency in patients on chronic corticosteroids, altered drug metabolism in the setting of hepatic involvement or malnutrition, and careful fluid management given the risk of dehydration from post-procedural nausea and vomiting. Anaesthetic assistance is regarded as essential for the safe performance of these procedures, a recommendation amplified in the IBD population by the additional layers of clinical complexity [10].

### 7.7. Early Post-Procedural Monitoring

Early post-procedural monitoring in IBD patients undergoing EBTs should be more intensive and structured than in the general bariatric endoscopy population. Nausea, vomiting, and abdominal pain are the most common adverse effects in the first days following both IGB placement and ESG, occurring in a large proportion of patients [11,45]. Aggressive antiemetic therapy, intravenous hydration, and proton pump inhibitor (PPI) prophylaxis should be initiated promptly, with prophylactic PPIs and continued lifestyle modification maintained throughout IGB therapy [45,66]. Diet progression should follow a structured protocol, typically clear liquids advancing gradually to pureed and then solid foods over two to four weeks, with particular attention to adequate protein intake to mitigate the risk of sarcopenia [8,66]. Fluid intake should be closely monitored, as dehydration from persistent vomiting may be more consequential in IBD patients with pre-existing electrolyte disturbances or renal vulnerability [10,63]. Severe or worsening abdominal pain, haematemesis, melaena, fever, signs of peritonitis, or inability to tolerate oral fluids beyond 48–72 h should prompt urgent evaluation.

### 7.8. Distinguishing Expected Post-Procedural Symptoms from Possible IBD Flare or Complication

Perhaps the most clinically challenging aspect of peri-procedural management in IBD is differentiating expected post-procedural symptoms from an IBD flare or a procedural complication. Nausea, vomiting, abdominal pain, bloating, and altered bowel habits are cardinal features of both the early post-procedural course and active IBD [7,11,22], and this ambiguity may lead to delayed recognition of flares, unnecessary device removal, or inappropriate escalation of IBD therapy. Clear symptom-monitoring protocols with predefined thresholds for biochemical reassessment should therefore be established before the procedure. Faecal calprotectin and serum C-reactive protein should be measured at baseline and repeated if symptoms persist beyond the expected window (typically 5–7 days for IGB, 7–14 days for ESG) or change in character [2,61]; a rising value in the context of persistent or atypical symptoms should prompt consideration of endoscopic evaluation to distinguish mucosal inflammation from device-related injury. Close communication between the bariatric endoscopist and the IBD specialist is indispensable, and patients should be counselled before the procedure about the expected symptom trajectory and the importance of reporting any deviation from it. No formal evidence-based algorithm for distinguishing post-procedural symptoms from IBD flares in this setting has been published; the approach outlined here rests on extrapolation from general IBD monitoring principles and expert reasoning, and represents an area in important need of prospective study.

## 8. Nutritional Risk and Longitudinal Follow-Up After Endoscopic Bariatric Therapy in Inflammatory Bowel Disease

### 8.1. Why Nutritional Follow-Up Is Critical in IBD

Patients with IBD occupy a uniquely vulnerable position on the nutritional spectrum, one that distinguishes them from the general obesity population undergoing endoscopic bariatric therapies (EBTs). They carry a heightened baseline risk for malnutrition, micronutrient deficiency, and sarcopenia, driven by chronic intestinal inflammation, malabsorption, decreased intake, hypermetabolism, and pharmacological treatment; covert deficits in lean mass may be unmasked only by targeted assessment, and ESPEN therefore recommends screening for malnutrition at diagnosis and regularly thereafter [5,8]. This narrower nutritional safety margin means that any intervention further restricting oral intake or altering absorption, as most EBTs do, carries a proportionally greater risk of precipitating clinically significant deficiency. The coexistence of obesity and malnutrition risk in the same patient demands that nutritional follow-up after EBTs be more intensive, more frequent, and more comprehensive than in the general bariatric endoscopy population [67].

### 8.2. Protein Intake and Preservation of Lean Mass

Preservation of lean body mass during weight loss is a critical therapeutic objective in IBD, given the high prevalence of sarcopenia and sarcopenic obesity. The ESPEN/UEG guideline reported that a systematic review found 42% of IBD patients to be sarcopenic; in an IBD population starting anti-TNF therapy, 4.9% of patients with obesity and 14.6% of those with overweight were sarcopenic, while 41.5% of those with normal weight also had sarcopenia [5]. With decreased muscle mass reported in 60% of adults with CD compared with healthy subjects, sarcopenic obesity is a defining feature of the changing IBD phenotype and should be assessed accordingly. Risk factors for excessive loss of lean body mass after bariatric procedures include reduced dietary quality, inadequate intake, altered nutrient absorption, and poor compliance with supplementation [68], and published sarcopenic-obesity monitoring algorithms should be applied, with therapy personalised to malnutrition risk, degree of obesity, tolerance, and patient preference [69]. Structured dietary counselling should therefore ensure a minimum protein intake of 1.0–1.5 g/kg ideal body weight per day during the weight-loss phase, adjusted by body-composition monitoring. No IBD-specific protein targets after EBTs have been published; those given here are extrapolated from general bariatric nutrition guidelines and should be validated in future studies.

### 8.3. Micronutrient Deficiencies: Iron, Vitamin B12, Folate, Vitamin D, Calcium, and Trace Elements

Micronutrient deficiencies are among the most consequential nutritional risks following EBTs, and the risk is amplified in IBD, where subclinical or overt deficiencies are often present at baseline. Obesity, IBD, and hypovitaminosis D are parallel and overlapping phenomena, and low serum vitamin D characterises obesity, IBD, and sarcopenic obesity [5]; its aetiology in IBD is multifactorial, reflecting malabsorption, inflammation, low dietary intake, low sun exposure, and corticosteroid therapy. Iron deficiency is similarly prevalent, driven by chronic blood loss, impaired absorption where duodenal or proximal jejunal inflammation is present, and the restrictive dietary patterns that follow EBT placement [8,67]. Vitamin B12 and folate deficiency may be exacerbated by reduced intake in the early post-procedural period, particularly after ESG, when patients are maintained on liquid diets for two to four weeks [7,70]. Few studies have assessed nutritional deficiencies following non-surgical endoscopic bariatric procedures, and such monitoring is not routinely performed in clinical practice [8]. Zinc, magnesium, and selenium, already at risk in IBD through chronic diarrhoea and malabsorption, may be further depleted by the caloric restriction EBTs impose. A structured micronutrient monitoring protocol extending well beyond the immediate post-procedural period is therefore required.

### 8.4. Suggested Follow-Up Strategy

No IBD-specific follow-up protocol after EBTs has been published. The framework proposed here, set out in full in Table 3, synthesises general bariatric endoscopy follow-up recommendations, IBD nutritional guidelines, and expert reasoning: contact at least twice within the first two weeks to assess tolerance of the liquid diet, hydration, and symptom trajectory, with electrolytes, renal function, and inflammatory markers (CRP, faecal calprotectin) to distinguish procedural symptoms from an IBD flare; comprehensive nutritional and body-composition assessment at one month; and review at three, six, and twelve months and at least annually thereafter, each visit combining dietary counselling, micronutrient testing, disease-activity assessment, and body-composition monitoring. Patients with gastrointestinal disease undergoing bariatric procedures should enter a follow-up programme designed for post-bariatric patients alongside follow-up of their primary disease [5], with regular blood tests to detect deficiencies early and allow prompt correction [71]. Supplementation should be individualised by disease location, extent, prior surgical history, and ongoing IBD therapy. This framework has not been validated in prospective studies and should be regarded as a starting point for clinical practice and future research rather than as an evidence-based guideline.

A proposed schedule for nutritional, body-composition, and disease-activity monitoring after EBT in patients with IBD is summarised in Table 3.

## 9. Reported Outcomes and Safety of Endoscopic Bariatric Therapies in Inflammatory Bowel Disease

### 9.1. Available IBD-Specific Evidence

The most striking feature of the current evidence landscape is its profound scarcity. To date, no randomised controlled trial, prospective cohort study, or even large retrospective case series has specifically evaluated the efficacy, safety, or disease-specific outcomes of any EBT, including IGBs and ESG, in a dedicated IBD population. The suggestion that EBTs may be effective and well tolerated in selected patients with IBD rests on extrapolation from general bariatric endoscopy data and limited indirect evidence rather than IBD-specific trials [2]; upper gastrointestinal IBD has been listed as a relative contraindication to IGB therapy without any accompanying outcome data from patients with IBD [7]. The most relevant indirect evidence comes from bariatric surgery: a nationwide propensity score-matched cohort of patients with IBD and obesity demonstrated improved IBD-related outcomes, including reduced hospitalisation and corticosteroid use, suggesting weight reduction itself may confer disease-specific benefit [57]. These surgical data cannot be directly extrapolated to endoscopic procedures, which differ in mechanism, magnitude of weight loss, and physiological impact. The absence of IBD-specific EBT outcome data is the single most important limitation of the current evidence base and must be explicitly acknowledged in any clinical discussion.

A recent systematic review and meta-analysis by Dean et al., pooling comparative studies of bariatric surgery outcomes in approximately 450,000 patients with and without IBD, substantially strengthens this indirect evidence base [72]. Weight loss was equivalent between the two groups, with no significant difference in the change in body mass index at one year, indicating that surgical weight-loss efficacy is preserved in IBD. Consistent with the disease-specific benefit reported by Stenberg et al. [57], bariatric surgery was associated with a significant decline in corticosteroid use among patients with IBD (RR 0.67, 95% CI 0.53–0.84), an effect observed in both ulcerative colitis and Crohn’s disease without preferential advantage to either subtype, and the direct comparison between subtypes revealed no significant difference in treatment escalation or de-escalation. Crucially for the present discussion, however, patients with IBD carried a higher risk of several adverse outcomes than non-IBD controls, including acute renal failure (RR 2.16, 95% CI 1.55–3.00), haemorrhage (RR 1.57, 95% CI 1.22–2.04), readmission (RR 1.56, 95% CI 1.17–2.08), and micronutrient deficiency; whereas, wound, anastomotic leak, thromboembolic, and bowel-obstruction rates did not differ significantly. Among procedures, Roux-en-Y gastric bypass was associated with more postoperative complications than sleeve gastrectomy (RR 2.21, 95% CI 1.43–3.41). Although these data derive from surgical rather than endoscopic interventions and cannot be extrapolated directly to EBTs, they reinforce two themes central to this review: that weight-loss intervention can favourably modulate IBD therapy requirements, and that patients with IBD remain at heightened risk of nutritional and other complications, underscoring the need for the intensive peri-procedural and nutritional monitoring detailed in Section 7 and Section 8.

### 9.2. Weight-Loss Outcomes

In the absence of IBD-specific data, weight-loss outcomes must be drawn from the general obesity literature and treated as indirect evidence; the figures for each modality are summarised in Table 1 and discussed in Section 4 [11,14,39]. In brief, ESG has demonstrated the most robust and durable outcomes, with the MERIT randomised controlled trial reporting a mean EWL of 49.2% at 52 weeks versus 3.2% for lifestyle control [73], and meta-analyses reporting TBWL of 15–20% at 12 months with weight loss sustained at 24 months and beyond [14,33,74]; ESG achieves greater and more durable weight loss than IGBs, though less TBWL than laparoscopic sleeve gastrectomy [12]. Whether these outcomes are reproducible in patients with IBD, who may have altered motility, nutrient absorption, and dietary tolerance, remains entirely unknown. The restrictive dietary phases following EBTs could plausibly be harder to adhere to in patients with IBD-related symptoms, attenuating efficacy, but this hypothesis is untested.

### 9.3. Metabolic Outcomes

EBTs in the general population have been associated with improvements in obesity-related metabolic comorbidities, including type 2 diabetes, dyslipidaemia, hypertension, and insulin resistance [35,43,75]; the MERIT trial reported that 80% of patients undergoing ESG experienced improvement in one or more metabolic comorbidities at 12 months [73], and IGBs improve glycaemic control and hepatic steatosis in the short term, although these benefits may not persist after removal [76]. Notable advances have been made in the metabolic improvements achieved by endoluminal therapies, some of which are now FDA-approved for metabolic disease rather than weight loss alone [43]. These potential benefits are of particular interest in IBD given the recognition that metabolic disorders increasingly affect this population with an expected negative impact on both disease entities [52]. However, no study has evaluated the metabolic effects of any EBT specifically in IBD, and it remains uncertain whether these improvements would be replicated or offset by disease-related metabolic derangements.

### 9.4. Effects on IBD Activity and Symptoms

Perhaps the most clinically consequential question, whether EBTs influence IBD disease activity beneficially or adversely, remains entirely unanswered by direct evidence. The rationale for benefit comes from the bariatric surgery literature, where surgery was associated with a reduced composite endpoint of IBD-related hospitalisation, corticosteroid initiation, new targeted therapy, and major IBD-related surgery, with an adjusted hazard ratio of 0.66 (95% CI 0.51–0.85) [57], suggesting weight reduction per se may favourably modulate disease course, possibly through reduced visceral adipose tissue-mediated inflammation and improved metabolic status. Conversely, bariatric interventions may promote intestinal dysbiosis and, in genetically predisposed patients, exaggerated intestinal inflammation, and de novo IBD should be considered in individuals with previous bariatric surgery who develop diarrhoea, anaemia, or excessive weight loss [54]. Whether these risks apply to EBTs, which are generally less physiologically disruptive, is unknown, and the symptom overlap discussed in Section 7.8 has not been addressed in any published study.

### 9.5. Quality of Life Outcomes

Quality of life (QoL) is critically important but understudied in this context. A systematic review and meta-analysis of endoscopic bariatric procedures in the general obesity population, including twenty studies of five different procedures and 876 patients, found IGB placement associated with a large improvement in QoL and mental health, concluding that EBTs may improve short-term QoL alongside weight loss and comorbidity improvement; these studies did not include patients with IBD, and their applicability to a population burdened by chronic gastrointestinal symptoms, fatigue, psychological distress, and the social impact of IBD is uncertain [77]. Patients with IBD frequently experience impaired QoL related to suboptimal disease control, and successful weight loss could plausibly improve both obesity- and IBD-related domains [58,59]; equally, the gastrointestinal side effects of EBTs could transiently worsen QoL in patients already symptomatic. No study has evaluated QoL outcomes of EBTs in IBD, and this represents an important gap for future research.

### 9.6. Main Limitations of the Current Evidence Base

The limitations of this evidence base are substantial and multifaceted: no study of any kind has evaluated an EBT in a defined IBD population, so all available evidence is either indirect or extrapolated from bariatric surgery; the general EBT literature itself suffers from heterogeneous design, follow-up often limited to 6–12 months, absent standardised endpoints, and underrepresentation of chronic gastrointestinal disease; and patients with upper gastrointestinal IBD are excluded from most EBT trials, whether explicitly or through contraindication lists, so that safety and efficacy remain unknown in the very patients who might be considered for them. The consequences of these limitations, and the specific questions they leave open regarding IBD-specific endpoints, drug interactions, and follow-up protocols, are examined in Section 10.

## 10. Knowledge Gaps and Future Directions

### 10.1. Why the Current Evidence Remains Insufficient

The central impediment to formulating evidence-based recommendations is the complete absence of prospective clinical studies designed to evaluate these procedures in this population. As set out in Section 9, all available evidence is either indirect or extrapolated from bariatric surgery, which differs fundamentally in mechanism, magnitude of physiological disruption, and long-term consequences; the suggestion that EBTs may be effective and well tolerated in selected patients with IBD was itself explicitly framed as extrapolation rather than a finding supported by disease-specific data [2]. Because patients with upper gastrointestinal IBD are excluded from most EBT trials, the very population for whom guidance is needed has been systematically underrepresented. This evidentiary vacuum prevents clinicians from making informed risk–benefit assessments and leaves patients without disease-specific safety and efficacy data.

It is worth interrogating why this gap persists, since the reasons are not merely a matter of insufficient attention. Several specific barriers can be identified. Clinically, most pivotal EBT trials have explicitly listed upper-gastrointestinal IBD as a contraindication or have implicitly excluded patients with chronic gastrointestinal disease through their eligibility criteria, so the very population of interest has been screened out by design; in addition, the substantial overlap between expected post-procedural symptoms and IBD activity complicates endpoint adjudication and discourages investigators from enrolling these patients. From a regulatory standpoint, endoscopic devices are approved for general obesity indications rather than for use in defined disease populations, and there is no IBD-specific approval pathway, which removes a commercial incentive for manufacturers to fund dedicated studies. Ethically and practically, deploying space-occupying devices or performing full-thickness gastric suturing in a population already predisposed to mucosal injury, malnutrition, and impaired healing raises a reasonable reluctance to expose such patients to incompletely characterised risks outside a carefully governed research setting. Recognising these barriers is important because each suggests corresponding remedy pragmatic registry designs that do not exclude IBD, IBD-aware adverse-event definitions, and collaborative investigator-initiated studies, as outlined in the research priorities below.

### 10.2. Key Unanswered Questions Regarding Patient Selection

No study has systematically evaluated which IBD patients are the best or worst candidates for specific EBTs. Critical unanswered questions include: What is the minimum duration of clinical and endoscopic remission required before an EBT can be safely offered? Does disease subtype (Crohn’s disease vs. ulcerative colitis) differentially affect procedural outcomes? How should disease phenotype—particularly stricturing, penetrating, or perianal behaviour in Crohn’s disease—influence the choice between intragastric balloons, endoscopic sleeve gastroplasty, and other modalities? What role should cross-sectional imaging play in preprocedural screening, and should small bowel evaluation be mandatory before IGB placement in all patients with Crohn’s disease? These questions cannot be answered by the existing literature and require dedicated prospective investigation.

### 10.3. Gaps in Safety Assessment

The safety profile of EBTs in IBD is entirely unknown. No study has reported adverse event rates, device-related complications, or IBD flare rates following any EBT in a defined IBD cohort. Theoretical concerns-including the risk of gastric ulceration at balloon contact sites in patients with subclinical gastric inflammation, suture dehiscence in patients on corticosteroids or immunosuppressants, and device-related obstruction in patients with unrecognised strictures-remain unvalidated. Wilson et al. demonstrated that IBD was associated with a more than two-fold increase in postoperative complications after bariatric surgery compared with non-IBD controls [78], raising the question of whether a similar risk amplification exists for endoscopic procedures. The interaction between EBTs and IBD pharmacotherapy-including the effects of anti-TNF agents, vedolizumab, ustekinumab, thiopurines, and methotrexate on procedural healing and infection risk-has not been investigated in any clinical context. Standardised adverse event reporting frameworks that capture both procedure-related and IBD-related complications are urgently needed.

### 10.4. Gaps in Nutritional and Metabolic Follow-Up Data

Very few studies have assessed nutritional deficiencies following non-surgical endoscopic bariatric procedures even in the general population [8], and in patients with IBD, who carry a heightened baseline risk of deficiency in iron, vitamin B12, folate, vitamin D, calcium, zinc, and protein, this gap is particularly consequential [5,61]. No study has evaluated the trajectory of micronutrient status, body composition, or sarcopenia risk following any EBT in an IBD cohort, nor quantified the risk of precipitating sarcopenic obesity through caloric restriction without adequate protein intake. The metabolic effects of EBTs, including changes in insulin resistance, adipokine profiles, and systemic inflammatory markers, are likewise unassessed in IBD despite the recognised interplay between visceral adiposity, metabolic dysfunction, and intestinal inflammation [52].

### 10.5. Need for Phenotype-Specific and Disease Activity-Specific Data

IBD is a heterogeneous group of disorders, and the safety and efficacy of EBTs are likely to vary substantially across disease subtypes, phenotypes, and activity states. Patients with ileal Crohn’s disease and inflammatory behaviour may face very different risks from those with extensive ulcerative colitis or stricturing ileocolonic disease. The impact of disease activity at the time of the procedure—including subclinical inflammation detectable only by biomarkers or endoscopy—on procedural outcomes is unknown. Future studies must stratify outcomes by IBD subtype, Montreal classification, disease activity (clinical, biochemical, and endoscopic), and concurrent pharmacotherapy to generate clinically actionable data.

### 10.6. Need for Comparative Studies Versus Pharmacotherapy and Surgery

No head-to-head study has compared EBTs with anti-obesity pharmacotherapy or bariatric surgery in patients with IBD. Pham et al. provided the only IBD-specific data on anti-obesity medications, demonstrating comparable weight loss and safety to non-IBD controls [79], but no analogous data exist for EBTs. Stenberg et al. and Desai et al. have provided disease-specific outcome data for bariatric surgery [57,80], establishing a benchmark against which EBTs should ultimately be compared. Comparative effectiveness studies-ideally randomised but at minimum well-designed observational cohorts with propensity score matching-are needed to determine the relative merits of pharmacotherapy, EBTs, and surgery across the spectrum of IBD phenotypes and obesity severity.

### 10.7. Priorities for Future Research

The most impactful next steps for advancing this field include the following. First, the establishment of multi-centre prospective registries that systematically capture EBT procedures performed in patients with IBD, recording baseline disease characteristics, procedural details, adverse events, IBD disease activity trajectories, nutritional outcomes, and body composition changes over a minimum of 12–24 months. Second, the conduct of prospective cohort studies—initially single-arm, with subsequent comparative designs-evaluating the safety and efficacy of ESG and IGBs in IBD patients stratified by disease subtype, phenotype, and activity state. Third, the development of standardised adverse event reporting frameworks that distinguish procedure-related complications from IBD flares and capture both short-term and long-term outcomes. Fourth, the integration of nutritional and metabolic endpoints—including micronutrient status, lean mass preservation, and inflammatory biomarkers—into all future EBT studies in IBD. Fifth, comparative effectiveness studies evaluating EBTs against anti-obesity pharmacotherapy and bariatric surgery in IBD-specific populations, with attention to both weight-loss and disease-specific outcomes. Sixth, and reflecting the increasingly metabolic framing of IBD, future work should harness emerging computational and molecular tools to move toward precision weight-management in this population. Metabolomic and lipidomic profiling, adipokine and microbiota-derived metabolite signatures, and analyses of bile-acid and short-chain fatty-acid metabolism may help to identify the patients in whom obesity is most metabolically active, and therefore most likely to benefit from weight reduction, as well as candidate biomarkers that track treatment response and nutritional risk after EBTs. In parallel, machine-learning and artificial-intelligence approaches that integrate multidimensional clinical, biochemical, body-composition, endoscopic, and metabolomic data could, in principle, support individualised candidate selection, risk prediction, and follow-up scheduling, provided that sufficiently large and well-phenotyped IBD-specific cohorts become available to train and externally validate such models. As a methodological example from outside the gastrointestinal field, the integration of multidimensional biomarkers using an artificial neural network has recently been demonstrated in other clinical settings [81], illustrating the type of modelling approach that could, in principle, be adapted to obesity and IBD once suitable datasets exist. These directions are at present aspirational and depend on the prospective, deeply phenotyped datasets called for above before they can be realised.

## 11. Conclusions

Obesity is an increasingly prevalent and clinically consequential comorbidity in patients with inflammatory bowel disease, exerting measurable effects on inflammatory burden, treatment response, surgical risk, and quality of life. Endoscopic bariatric therapies, particularly intragastric balloons and endoscopic sleeve gastroplasty, are minimally invasive, potentially reversible, and efficacious weight-loss interventions in the general obesity population, and their favourable safety profile, avoidance of permanent anatomical alteration, and repeatability make them conceptually attractive for patients with IBD who require weight reduction but face unique risks from more invasive approaches.

However, the current evidence base for EBTs in IBD is profoundly limited. No prospective trial, large retrospective cohort, or standardised registry has specifically evaluated the safety, efficacy, or disease-specific outcomes of any EBT in a defined IBD population. All clinical reasoning in this domain is therefore derived from indirect evidence in the general bariatric endoscopy literature, extrapolation from bariatric surgery data in IBD, and expert opinion. This evidentiary gap must be acknowledged transparently in all clinical discussions with patients.

What can be stated with reasonable confidence is that the safe application of EBTs in IBD demands rigorous, individualised, multidisciplinary evaluation: assessment of disease phenotype, confirmation of sustained clinical and endoscopic remission, review of prior surgical history and resultant anatomy, optimisation of baseline nutritional status and body composition, and scrutiny of current pharmacotherapy for its impact on healing and monitoring, followed by structured longitudinal follow-up encompassing nutritional surveillance, disease-activity monitoring, and body-composition assessment. The practical basis for each of these steps is set out in Section 6, Section 7 and Section 8 and Table 2 and Table 3.

Looking forward, the convergence of rising obesity prevalence in IBD and the expanding availability of endoscopic bariatric technologies creates both an opportunity and an obligation. Multi-centre prospective registries, phenotype-stratified cohort studies, and comparative effectiveness trials are urgently needed to transform the current landscape of extrapolation and expert reasoning into one of evidence-informed clinical practice, ensuring that patients with IBD and obesity receive care that is both effective and safe.

## Figures and Tables

**Table 1:** Endoscopic bariatric therapies and their principal considerations in patients with inflammatory bowel disease (IBD). Efficacy figures are derived from general-population studies; no endoscopic bariatric therapy has been evaluated in a dedicated IBD population.

Therapy	Mechanism	Reported Efficacy (General Population)	Evidence Base	Principal IBD-Specific Considerations
Intragastric balloon (IGB)	Space-occupying device; reduces functional gastric volume, promotes early satiety, delays gastric emptying	~10–15% TBWL and 25–50% EWL at 6 months; weight regain common after removal	Randomised trials and large cohorts; longest-established EBT	Upper-GI IBD is a relative contraindication; high incidence of nausea/vomiting may mimic a flare; risk of distal migration and obstruction at strictures; requires intact gastric mucosa
Endoscopic sleeve gastroplasty (ESG)	Full-thickness gastric suturing reducing gastric volume by ~70%	15–20% TBWL at 12 months, durable to ≥24 months; MERIT trial 49.2% EWL at 52 weeks	Strongest EBT evidence base (RCT and meta-analyses)	Full-thickness suturing raises concern with corticosteroid/immunosuppressant exposure and transmural disease; requires a healthy gastric wall; post-procedural symptoms overlap with flare
POSE/POSE 2.0	Endoscopic gastric plication (fundus or distal stomach)	~12.7–13.5% TBWL; EWL ~45–49%; lower than ESG	Smaller evidence base; not FDA-approved for bariatric use	Limited data; same gastric-wall integrity and symptom-overlap concerns as ESG
Aspiration therapy	Percutaneous gastrostomy-based partial aspiration of gastric contents after meals	Clinically significant TBWL in the general population	Approved; niche use	Gastrostomy site raises wound-healing and infection concerns in immunosuppressed patients; not studied in IBD
Duodenal–jejunal bypass liner (DJBL)	Endoluminal small-bowel sleeve diverting nutrients past the proximal small bowel	Weight and metabolic benefit in the general population	Limited; withdrawn in some markets	Particular caution in CD with upper-GI or small-bowel involvement; hazardous with strictures, prior resection, or short bowel; malabsorptive mechanism compounds IBD nutritional risk
Duodenal mucosal resurfacing (DMR)	Hydrothermal ablation of duodenal mucosa (primarily metabolic)	Mainly metabolic (glycaemic) benefit	Investigational; limited	Cautioned or contraindicated with duodenal CD or upper-GI involvement; mucosal ablation of inflamed or at-risk duodenum is hazardous

TBWL, total body weight loss; EWL, excess weight loss; EBT, endoscopic bariatric therapy; CD, Crohn’s disease; FDA, U.S. Food and Drug Administration; RCT, randomised controlled trial; GI, gastrointestinal.

**Table 2:** Proposed candidacy, cautions, and red flags for endoscopic bariatric therapies (EBTs) according to inflammatory bowel disease (IBD) features, with the level of supporting evidence. No formal evidence-based eligibility criteria for EBTs in IBD have been established; all entries reflect indirect evidence or expert consensus and require prospective validation.

IBD Feature	Suggested Implication for EBT Candidacy	Basis/Level of Support
Sustained clinical and endoscopic remission (≥3–6 months)	Favourable; regarded as a pre-requisite before EBT	Extrapolated from bariatric surgery data and general endoscopic principles; expert consensus
Active mucosal inflammation, recent flare, or raised CRP/faecal calprotectin	Defer until disease control is re-established	Expert consensus; extrapolated
Ulcerative colitis in remission, no upper-GI pathology	More straightforward candidate for gastric-based EBTs	Indirect (surgical) evidence and expert reasoning
Gastric or duodenal Crohn’s disease (upper-GI CD)	Relative or absolute contraindication to gastric-based EBTs and to DJBL/DMR	Extrapolated; expert consensus
Stricturing (B2) behaviour	Caution or avoid; risk of device impaction or obstruction (especially IGB)	Extrapolated; expert consensus
Penetrating (B3) or fistulizing disease	Caution or avoid	Expert consensus
Prior extensive small-bowel resection or short bowel	Avoid malabsorptive small-bowel EBTs	Expert reasoning; extrapolated
Severe malnutrition, active sarcopenia, or uncorrected micronutrient deficiency	Correct before any EBT is undertaken	ESPEN/UEG guidance (adapted)
Current or recent high-dose corticosteroid therapy	Wean to lowest effective dose or transition to steroid-sparing agents first	Extrapolated from surgical principles; expert consensus
Inability to ensure structured multidisciplinary follow-up	Relative contraindication	Expert consensus

CRP, C-reactive protein; CD, Crohn’s disease; GI, gastrointestinal; IGB, intragastric balloon; DJBL, duodenal–jejunal bypass liner; DMR, duodenal mucosal resurfacing; ESPEN, European Society for Clinical Nutrition and Metabolism; UEG, United European Gastroenterology.

**Table 3:** Proposed nutritional, body-composition, and disease-activity monitoring schedule after endoscopic bariatric therapy (EBT) in patients with inflammatory bowel disease (IBD). This framework is synthesised from general bariatric-endoscopy follow-up, IBD nutritional guidelines, and expert reasoning, and has not been validated in IBD-specific prospective studies.

Timepoint	Nutritional and Body-Composition Assessment	IBD Disease-Activity Assessment	Purpose
Baseline (pre-procedure)	CBC; iron studies (ferritin, transferrin saturation); vitamin B12; folate; 25-hydroxyvitamin D; calcium; magnesium; zinc; thiamine; albumin/prealbumin; body composition (CT/MRI or DXA/BIA)	CRP, ESR, faecal calprotectin; confirm remission	Establish baseline and correct deficiencies before intervention
0–2 weeks	Electrolytes, renal function, hydration and oral-tolerance review	CRP and faecal calprotectin if symptoms persist or change	Detect dehydration; distinguish expected procedural symptoms from a flare
1 month	Full micronutrient panel; body composition (BIA or imaging) for lean-mass preservation	Disease-activity review	Detect early deficiency and incipient sarcopenia
3, 6, and 12 months, then at least annually	Micronutrient panel; body composition; dietary counselling targeting protein ≥1.0–1.5 g/kg ideal body weight per day	Clinical and biochemical activity; endoscopy if indicated	Long-term nutritional surveillance and preservation of lean mass

CBC, complete blood count; CRP, C-reactive protein; ESR, erythrocyte sedimentation rate; CT, computed tomography; MRI, magnetic resonance imaging; DXA, dual-energy X-ray absorptiometry; BIA, bioelectrical impedance analysis.

## Data Availability

No new data were created or analyzed in this study.

## Abbreviations

The following abbreviations are used in this manuscript:

**Abbreviation**	**Full term**
AGA	American Gastroenterological Association
ASGE	American Society of Gastrointestinal Endoscopy
BMI	Body mass index
CD	Crohn’s disease
CRP	C-reactive protein
CTE	Computed tomography enterography
EBT	Endoscopic bariatric therapy
ESG	Endoscopic sleeve gastroplasty
ESGE	European Society of Gastrointestinal Endoscopy
ESPEN	European Society for Clinical Nutrition and Metabolism
EWL	Excess weight loss
GLP-1 RA	Glucagon-like peptide-1 receptor agonist
IBD	Inflammatory bowel disease
IGB	Intragastric balloon
MRE	Magnetic resonance enterography
NF-κB	Nuclear factor kappa-light-chain-enhancer of activated B cells
NSAIDs	Nonsteroidal anti-inflammatory drugs
POSE	Primary obesity surgery endoluminal
PPI	Proton pump inhibitor
QoL	Quality of life
RYGB	Roux-en-Y gastric bypass
SG	Sleeve gastrectomy
TBWL	Total body weight loss
TNF-α	Tumour necrosis factor-alpha
UC	Ulcerative colitis
UEG	United European Gastroenterology
